# Targeting EBV-associated gastric cancer by lytic induction therapy with nanatinostat

**DOI:** 10.1016/j.tvr.2026.200346

**Published:** 2026-06-27

**Authors:** Man Wu, Shin Yee Hui, Andrew Skora, Yuk Yu Chan, Grace Tin-Yun Chung, Lili Li, Qian Tao, Dajiang Guo, Christopher W. Dawson, Lawrence S. Young, Chi Man Tsang, Ayman El-Guindy, Kwok Wai Lo

**Affiliations:** a Department of Anatomical and Cellular Pathology, Prince of Wales Hospital, The Chinese University of Hong Kong, Hong Kong SAR, China; b Viracta Therapeutics, IC., San Diego, CA, United States; c Department of Clinical Oncology, Prince of Wales Hospital, The Chinese University of Hong Kong, Hong Kong SAR, China; d State Key Laboratory of Translational Oncology, Sir YK Pao Centre for Cancer, The Chinese University of Hong Kong, Hong Kong SAR, China; e Warwick Medical School, University of Warwick, Coventry CV4 7AL, UK

**Keywords:** Epstein-barr virus, Gastric cancer, Nanatinostat, HDAC inhibitor, Lytic induction therapy

## Abstract

Latent Epstein-Barr virus (EBV) infection is associated with multiple lymphoid and epithelial cancers in humans. Targeting EBV through lytic induction therapy represents a potential strategy for treating virus-associated malignancies, such as EBV-associated gastric cancer (EBVaGC). Despite its classification as a distinct gastric cancer subtype, precision therapeutic strategies for EBVaGC remain vastly underexplored. In a recent clinical study, the combination of an orally administered histone deacetylase (HDAC) inhibitor, nanatinostat (NSTAT) with valganciclovir (VGCV), showed promise as a lytic induction therapy for EBV-positive lymphoma. In this study, we evaluated the activity of NSTAT to induce EBV lytic reactivation and the therapeutic efficacy of NSTAT-based lytic induction therapy in two representative EBVaGC cell lines *in vitro* and *in vivo.* NSTAT efficiently induced the expression of EBV immediate-early, early and late genes in EBVaGC cells. In addition, NSTAT treatment also promoted global histone acetylation and suppressed c-MYC and BCL2 expression, leading to cell cycle arrest and cell death in the EBVaGC tumor cells. Importantly, this study demonstrated the potent antitumor efficacy and safety of combined NSTAT and ganciclovir (GCV) treatment in both *in vitro* and *in vivo* preclinical EBVaGC models.

## Highlights

• NSTAT is an orally bioavailable, Class I-HDACi targeting EBVaGC in nanomolar range.
• NSTAT effectively induced lytic reactivation in patient-derived EBVaGC models.
• NSTAT suppressed c-MYC signaling and altered cell cycle regulations in EBVaGC cells.
• Potent *in vivo* inhibitory effect of lytic induction therapy with NSTAT in EBVaGC.

## Graphical abstract

**Figure:**
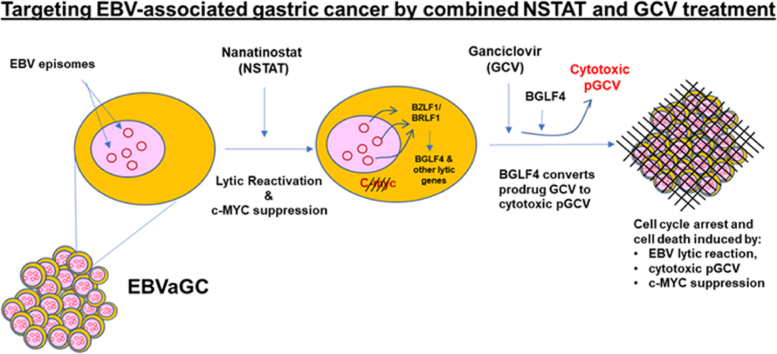

## Abbreviations

EBV: Epstein-Barr virus
EBVaGC: EBV-associated gastric cancer
GCV: ganciclovir
HAT: histone acetyltransferases
HDAC: histone deacetylase
HDACi: histone deacetylase inhibitor
IHC: immunohistochemical staining
NSTAT: nanatinostat
RISH: RNA *in situ* hybridization
SAHA: suberoylanilide hydroxamic acid

## 1 Introduction

Epstein-Barr virus (EBV) is an oncogenic virus that contributes to the development of multiple lymphomas and epithelial malignancies in humans, including Burkitt lymphoma (BL), Hodgkin lymphoma, nasopharyngeal carcinoma (NPC) and gastric cancer. Clonal EBV genomes exist in every tumor cell of these EBV-associated cancers and drive oncogenesis via various latent gene expression programs (type I, II or III latency). Latent genes, including *EBNA* family genes, *LMP1/2, EBER1/2*, viral lncRNAs and microRNAs, contribute to tumorigenesis through multiple mechanisms such as the activation of oncogenic signaling pathways, promotion of immune evasion, induction of genomic instability and alteration of the epigenome [1]. Among EBV-positive cancers, More than 40% of all EBV-positive cancers are EBV-associated gastric cancer (EBVaGC), for which the annualglobal incidence rate range from 75,000 to 90000 [1,2]. Unlike NPC, which is consistently linked to EBV, only approximately 10% of gastric cancers are associated with EBV infection. All EBVaGCs except the rare lymphoepithelioma-like carcinoma subtype expresses a restricted latency I transcriptional program. In EBVaGC, latent genes such as *EBERs*, *EBNA1, LMP2* and *BARTs* play key roles in tumor development. The Cancer Genome Atlas (TCGA) study has defined EBVaGC as one of four distinct gastric cancer subtypes based on its association with EBV infection and unique molecular features, including genome-wide DNA hypermethylation, elevated programmed death ligand 1(PD-L1)/PD-L2 expression and frequent *PIK3CA* and *ARID1A* mutations [[2], [3], [4]]. Although recent advances in immunotherapy and biomarker-driven targeted therapies have improved the clinical management of several subgroups of gastric cancer, a specific treatment strategy targeting EBV-positive gastric cancer remains lacking.

The persistent presence of clonal EBV genome and the requirement of multiple viral gene products for malignant transformation suggest that EBV-targeted therapies could improve the clinical outcomes of EBVaGC patients [4,5]. Various therapeutic strategies that target EBV-associated cancers by either inhibiting EBV latency or inducing the viral lytic cycle have been developed over the past two decades. Some recent studies have also reported the clinical responses of patients with EBV-associated malignancies to these targeted therapies [[6], [7], [8], [9], [10], [11], [12]]. Among the EBV-encoded genes, *EBNA1* is consistently expressed in all EBV latency programs and plays crucial roles in EBV episomes replication and maintenance. VK-2109, an EBNA1 inhibitor that blocks the EBNA1-DNA interaction, has shown potent antitumor effects in both *in vitro* and *in vivo* preclinical models of NPC and EBVaGC [[12], [13], [14]]. A phase I clinical study demonstrated that VK-2019 treatment was well tolerated and resulted in a partial response or stable disease in eight of 23 patients with advanced EBV-positive NPC [12]. EBV lytic induction therapies targeting the viral latent-lytic switch have also been explored over the last three decades. In EBV-infected cells, the switch from latency to the lytic cycle is controlled by the immediate-early lytic proteins, BZLF1 and BRLF1. These proteins induce the transcription of early lytic genes and drive lytic progression, leading to new virion production and disrupting EBV-infected cells [[6], [7], [8]]. Using the CRISPR-Casilio activator system, we recently demonstrated that inducing endogenous *BZLF1* was sufficient to induce *in vivo* cytolytic and antitumor effects in EBV-positive NPC and EBVaGC tumors [15**]**. During lytic reactivation, the early lytic protein BGLF4, a serine/threonine protein kinase, is expressed and can phosphorylate the ganciclovir (GCV) prodrug to convert it into its cytotoxic form. Phosphorylated GCV inhibits viral DNA replication, induces host cell-cycle arrest and apoptosis, and facilitates the bystander killing effect to eradicate nearby cancer cells [[6], [7], [8]]. The therapeutic efficiencies of several small-molecule lytic inducers, such as histone deacetylase (HDAC) inhibitors in combination with GCV or valganciclovir have been evaluated in clinical trials for EBV-associated cancers. Several such trials have demonstrated the therapeutic benefits of these treatments in the context of EBV-positive lymphoid malignancies [9]. A recent phase 1b/2 study of the orally bioavailable HDAC inhibitor (HDACi), nanatinostat (NSTAT) in combination with valganciclovir (VGCV) yield high overall response rate (60%) and complete response rate (27%) in a panel of EBV-positive lymphoid malignancies [16].

Histone acetylation plays a crucial role in transcriptional regulation by modifying the structure of chromatin and its accessibility to transcription factors. This modification is dynamically regulated by two enzyme classes: histone acetyltransferases (HATs) and HDACs. Eighteen HDACs that catalyze the removal of acetyl groups from the lysine residues of both histone and nonhistone proteins have been identified in human cells [17]. HDAC inhibitors are a class of small molecular drugs that bind to HDACs and block their deacetylation ability. The efficacy of HDAC inhibitors for treating lymphoid malignancies and solid tumors has been investigated in clinical trials [17,18]. In addition to inducing EBV lytic reactivation, HDAC inhibitors may restore cellular acetylation homeostasis and the expression of tumor suppressors by modifying their cis-regulatory element activity in EBV-associated cancers. The antitumor effects of HDAC inhibitors are believed to depend on the ability to induce EBV lytic reactivation and interfere with host oncogenic pathways.

In our pilot study of a panel of common HDAC inhibitors, we found that only NSTAT, TSA and romidepsin exhibited nanomolar-range IC_50_ value in the native EBVaGC cell lines SNU719 and YCCEL1 (Supplementary Fig. S1). Although romidepsin exerted potent cytotoxic effects against these cell lines, it failed to induce ZEBRA expression in YCCLE1 cells (Supplementary Fig. S2). Notably, the pan-HDAC inhibitors SAHA and TSA also failed to induce ZEBRA expression in these EBVaGC cell lines. NSTAT is an orally bioavailable, highly selective class I HDAC inhibitor that primarily targets HDAC1, HDAC2 and HDAC3. Given the promising results from a clinical study of NSTAT-based lytic induction therapy for EBV-positive lymphoid malignancies, in this study we explored its lytic induction ability and therapeutic efficacy in preclinical models of EBVaGC. Specifically, we evaluated the therapeutic effects of NSTAT in relevant native EBVaGC cell lines. In addition to efficiently inducing ZEBRA and activating downstream EBV lytic proteins, NSTAT suppressed c-MYC and BCL2 expression, which led to cell cycle arrest and apoptosis in EBVaGC cells. We further used *in vivo* cell-derived xenograft (CDX) preclinical models to demonstrated the safety and potent therapeutic efficacy of NSTAT in combination with GCV for the treatment of EBVaGC.

## 2 Materials and methods

### 2.1 Cell cultures and drugs

Three native EBVaGC cell lines, SNU719, YCCEL1 and NCC24, were used in this study. YCCEL1, a generous gift from Professor Quen Tao, was maintained in our laboratories. SNU719 and NCC24 were obtained from the Korean Cell Line Bank (Seoul, Republic of Korea). EBV-positivity was detected in only 10-20% of NCC24 cells (Supplementary Fig. S3) [[19], [20], [21]]. We also included AGS-BX1, an EBV-reinfected gastric cancer cell line maintained in our laboratory [22]. The cells were cultured in RMPI-1640 medium supplemented with 10% fetal bovine serum at 37 °C and 5% CO_2_. All the cell lines used in this study were authenticated using short tandem repeat (STR) profiling and *EBER in situ* hybridization. NSTAT was provided by Viracta Therapeutics (San Diego, CA, USA). The HDAC inhibitors used in this study included sodium butyrate (NaB), TSA, SAHA and romidepsin (MedChemExpress, Monmouth Junction, NJ, USA). The antiviral drug GCV was purchased from Roche (Basel, Switzerland).

### 2.2 Quantitative RT-PCR assay

Total RNA was extracted from the cells using TRIzol reagent (Thermo Fisher Scientific, Waltham, MA, USA). The extracted RNA was reverse transcribed into complementary DNA using the RT Regent Kit with gDNA Eraser (TaKaRa, Kyoto, Japan). Quantitative real-time PCR with the SYBR Green master mix (Thermo Fisher Scientific) was performed to determine the expression of EBV lytic genes as described previously [15].

### 2.3 Western blot analysis

Protein extracts were prepared from cell pellets using RIPA lysis buffer supplemented with protease inhibitor (Roche, Basel, Switzerland). Equal amounts of proteins in each extract were separated by sodium dodecyl-sulfate polyacrylamide gel electrophoresis (SDS-PAGE) and transferred onto 0.45 μm nitrocellulose membranes. After blocking, the membranes were incubated with the appropriate primary antibodies, including anti-BZLF1/ZEBRA (BZ1, Santa Cruz Biotechnology, Dallas, TX, USA; dilution = 1:1000), anti-Rta (8C12, Argene, Varilhes, France; 1:1000), anti-EA-D (1108-1, Santa Cruz Biotechnology; 1:1000); anti-BGLF4 (1:1000), VCAp18 (#PA1-73003, Invitrogen; 1:500); anti-c-MYC (#A1309, ABclonal, Woburn, USA; 1:1000); anti-BCL2 (Clone 124, DAKO, Santa Clara, CA, USA; 1:1000); anti- HDAC1 (#5356, Cell Signaling; Boston, MA, USA; 1:2000); anti-HDAC2 (#5113, Cell Signaling; 1:2000); anti-HDAC3 (#3949, Cell Signaling; 1:2000); anti-H3K27ac (#ab4729, Abcam, Cambridge, MA USA; 1:4000); anti-Vinculin (#V9131, Sigma, St. Louis, MO, USA; 1:10,000), anti-Actin (13E5, Cell Signaling; 1:4000) and anti-GAPDH (#2118, Cell Signaling; 1:4000) antibodies. After washing and incubation with secondary antibody, signals on the blots were detected using the ChemiDoc Image system (Bio-Rad) [15].

### 2.4 Immunohistochemical staining

Immunohistochemical staining was used to detect expression of the EBV lytic proteins ZEBRA and EA-D in the tumor sections of EBV-positive xenografts. In brief, 4-μm sections of formalin-fixed paraffin-embedded tumors were dewaxed, rehydrated, and washed with water. After antigen retrieval, the samples were incubated with one of the following antibodies: anti-BZLF1/ZEBRA (BZ1, Santa Cruz Biotechnology; 1:100), anti-EA-D (1108-1, Santa Cruz Biotechnology; 1: 100), anti-c-MYC (#5605, Cell Signaling; 1:100), anti-CD68 (#ab125212, Abcam; 1:150), anti-mNKp46 (#AF2225, R&D Systems; 1:100) or anti-Ki67 (#610968, BD Pharmingen; 1:1000). After incubation with a horseradish peroxidase-labeled secondary antibody, the sections were developed with 3,3′-diaminobenzidine, and counterstained with hematoxylin (Sigma) [15].

### 2.5 RNAscope RNA *in situ* hybridization (RISH)

*BGLF4* and *EBER1* expression was detected using RNAscope 2.0 RISH assays and relevant probes (Advanced Cell Diagnostics, USA). Formalin-fixed paraffin-embedded (FFPE) tissue sections or cell blocks (4 μm) were deparaffinized in xylene and then dehydrated in an aqueous ethanol series. The tissue sections were treated with DNase, after which *BGLF4*- and *EBER1*-specific probes were hybridized to the tissue sections, followed by incubation with preamplifier, amplifier and labeled probes according to the manufacturer's instructions [15]. Representative images of the stained section were acquired. Cells expressing EBV lytic transcripts (*BGLF4*) in tumor sections from mice treated with NSTAT and controls were evaluated.

### 2.6 Detection of infectious EBV particles

The culture supernatants from SNU719 or YCCEL1 cells treated with NSTAT alone and in combination with GCV for 48 h were harvested and centrifuged at 260 g for 5 min. Cell debris was removed by filtering the supernatants through a 0.45-μm cellulose acetate filter. The filtered supernatants were then mixed with equal volumes of 16% PEG800-NaCl buffer and incubated overnight at 4 °C. EBV particles were pelleted by centrifugation at 1200 g for 1 h at 4 °C. After discarding the supernatant, the pellet was resuspended in RPMI-1640 medium supplemented with 10% fetal bovine serum at a volume corresponding to 1/25 of the original supernatant volume. These procedures increased the EBV concentration in the culture supernatant by 25 fold. EBV-negative Akata cells were incubated with the concentrated supernatant for 3 days. After incubation, the Akata cells were harvested, and EBV latent gene expression was determined via qRT-PCR analysis [15].

### 2.7 Flow cytometric analysis

After trypsinization and washing with phosphate-buffered saline (PBS), NSTAT-treated SNU719 and YCCEL1 cells were fixed with 4% paraformaldehyde and permeabilized with 0.1% TritonX-100 in PBS. The cells were stained with an Alexa-647-conjugated mouse anti-ZEBRA antibody (BZ1, Santa Cruz Biotechnology; 1:100) and Alexa-594-conjugated anti-EA-D antibody (1108-1, Santa Cruz Biotechnology; 1:100), then analyzed using a BD LSRFortessa Cell Analyzer (Becton Dickinson, Franklin Lakes, NJ, USA). The data were analyzed using FlowJo software, version 10 (FlowJo, LLC, Ashland, OR, USA). Briefly, total cells were first gated to exclude obvious debris based on forward scatter (FSC-A) and side scatter (SSC-A) characteristics with voltages optimized for each cell type. After that, the overall ZEBRA positive or EA-D positive cell populations were identified using the APC channel (for Alexa647-conjugated ZEBRA) or the FITC channel (for Alexa488-conjugated EA-D), respectively, against FSC to define positive gating boundaries.

### 2.8 Cell cycle analysis

Cells were detached from the culture plates using trypsin, washed with cold PBS, and fixed in 70% ethanol at 4 °C overnight. After washing with PBS, the cells were incubated with propidium iodide (PI; 1 μg/mL; Invitrogen, P3566) and RNase (10 μg/mL; Roche) for 30 min. The cells were washed after RNase treatment. For each sample, 10^4^ cells were subjected to cell cycle analysis on a FACSCalibur Flow Cytometer (BD Biosciences) to detect the DNA content. FlowJo software was used for data analysis.

### 2.9 Live/Dead cell viability assay

*Dead cell*s were determined using PI (Invitrogen, P3566), with Hoechst 33,342 (#H1399, ThermoFisher) counter staining as previously described [23]. Briefly, valid cells were gated using nuclear stain signals with a fixed exposure time on an ImageXpress Micro Confocal High Content Imaging system (Molecular Devices). A minimum average of 1000 cells were captured per single well at 10x magnification in a 96-well format, which was used for the analysis. The target average intensity was used to determine the cut-off values for PI signal intensity. *Dead cell*s are defined here as valid cells with PI signals.

### 2.10 Cell viability determination

Approximately 10^4^ cells per well were seeded in per 100-μL volume in 96-well plates the day before HDAC or control treatment. Following treatment, the medium was refreshed, and 10 μL of CCK-8 reagent (Dojindo Molecular Technologies, Rockville, MD, USA) were added to each well to determine cell viability. After incubation at 37 °C for 3–4 h, the absorbance in each well was measured at 450 and 650 nm using a 96-well SpectraMax plate reader (Molecular Devices, San Jose, CA, USA). Cell growth inhibition in each well was calculated as follows: (viability_control_ -viability_drug_)/viability_control_ × 100%. Each sample was analyzed in triplicate.

### 2.11 RNA sequencing

Total RNA was extracted from NSTAT-treated EBV-positive gastric cancer cells using TRIzol reagent (Invitrogen); subsequently, the RNA profiles of the cells were evaluated. Stranded specific poly(A) enriched RNA sequencing libraries were prepared for NSTAT-treated cells. For samples from CDX mice model, stranded specific RNA sequencing libraries with DNase I treatment and rRNA and globin depletion were generated. Next-generation sequencing (150bp, paired-end) was performed on an Illumina HiSeq 1500 sequencing system (Illumina, San Diego, CA, USA). After removing adapter sequences and low-quality reads, the remained reads were aligned and annotated to the human reference genome (GRCh38) and EBV genome (chrEBV_Akata_inverted) using Hisat2 (2.2.0) with the “--rna-strandness RF” parameter and StringTie (1.3.6). Downstream analyses were performed on the R platform (v4.5.0). Differentially expressed genes (DEGs) between the control and NSTAT treated samples were identified using the DEseq2 package (1.48.2) with a false discovery rate cut-off below 0.05 [24]. Volcano plots were generated using the ggplot 2 package (3.4.1). Gene Set Enrichment Analysis (GSEA) of the RNA sequencing samples was performed using the clusterProfiler package (3.21) with predefined Hallmark gene sets [25,26].

### 2.12 Chromatin immunoprecipitation (ChIP)-seq data analysis

Chromatin from both fixed NSTAT-treated and control cells was prepared using a truCHIP Chromatin Shearing Kit (Covaris, Woburn, USA), then broken into 200–500 bp DNA fragments using a Covaris S220 Focused-ultrasonicator (Covaris). The resulting protein–DNA complexes were immunoprecipitated using 5 μg of an anti-H3K27ac (#ab4729, Abcam, Cambridge, USA) on a rotator at 4 °C overnight and then purified using magnetic beads (26,162, Pierce; Thermo Fisher). After washing, crosslink reversal and DNA purification were performed, and 8 ng of immunoprecipitate and input DNA were used to construct each Illumina sequencing library according to the manufacturer's protocol (Kapa Hyper Prep Kit, KK8504, Roche). Each library was sequenced on a Nextseq 500 platform (Illumina) to obtain 150 base paired-end reads. The ENCODE histone ChiP-seq pipeline (v2.0) was applied for alignment and peak calling (GitHub - ENCODE-DCC/chip-seq-pipeline2: ENCODE ChIP-seq pipeline). DESeq2 was used to analyze the differentially accessible regions.

### 2.13 *In vivo* study

SNU719 (2x10^6^) and YCCEL1 (1x10^7^) cells were resuspended in a total 150-μL volume of culture medium and matrix gel. The cells were subcutaneously injected into the right flanks of female NOD SCID mice at 5-6 weeks old, which were randomly assigned to different experimental groups. To evaluate the lytic reactivation of NSTAT *in vivo*, mice administered either YCCEL1 or SNU719 CDXs were fed NSTAT once (15 mg/kg) or twice (7.5 mg/kg) daily for 3 or 6 days, respectively. To evaluate the therapeutic efficacy of NSTAT in mice with EBV-positive gastric cancers, mice were fed NSTAT (10 mg/kg) via oral gavage daily alone or with GCV (30 mg/kg, intraperitoneal injection) daily once the tumors had reached an approximate volume of 100 mm^3^. The control condition was an equal volume of PBS. Tumor volumes were calculated using formula 0.5 × *l* × *w*^2^, where *l* and *w* represent the tumor length and width, respectively. The mice were weighed and the tumor size were monitored every 3-4 days until the tumors in the control group exceeded a volume of 1000 mm^3^. The maximal tumor size permitted by the University Animal Experimentation Ethics Committee (AEEC) of the Chinese University of Hong Kong is 2000 mm^3^. Serum samples and organs, including the heart, lung, liver, spleen and kidney, were collected at the end of the experiment.

### 2.14 TUNEL assay

Four-μm sections of FFPE tumor tissues were deparaffinized in xylene, dehydrated in an aqueous ethanol series and subjected to a TUNEL assay using a TUNEL assay kit (#ab206386, Abcam) according to the manufacturer's protocol.

### 2.15 Statistical analysis

At least three independent replicates of *in vitro* experiments were performed. The statistical significance of the data was analyzed using Student's t-test or a one-way analysis of variance (ANOVA). A p-value <0.05 was considered statistically significant. *In vitro* results are performed as means ± SD. *In vivo* results are presented as the means ± standard errors of the means (SEM). All statistical analyses were performed using GraphPad Prism 8.0 software.

## 3 Results

### 3.1 NSTAT efficiently induces EBV lytic reactivation and cell death in EBVaGC

**Fig. 1:**
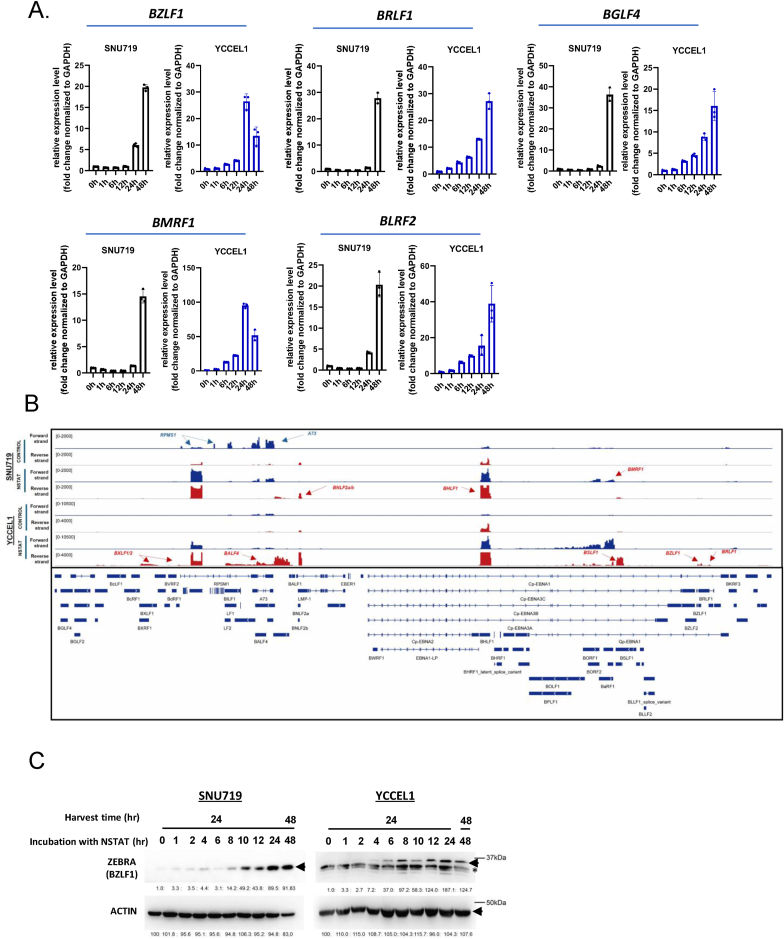
**Nanatinostat (NSTAT) reactivated EBV lytic genes in EBVaGC.** (A) Expression of the lytic genes *BZLF1*, *BRLF1*, *BGLF4*, *BMRF1* and *BLRF2* in NSTAT treated SNU719 and YCCEL1 cells after 1, 6, 12, 24, or 48h was determined by quantitative RT-PCR. The data are presented as the mean ± SD. (B) Representative EBV transcriptome profiles of NSTAT treated SNU719 and YCCEL1 cells illustrate the induction of multiple EBV lytic genes after 24h of treatment. EBV lytic genes are indicated by red arrows. (C) *Western blot*ting revealed endogenous expression of the immediate-early lytic protein ZEBRA in SNU719 and YCCEL1 cells treated with NSTAT for 0 to 24 h (Arrow: ZEBRA or ACTIN; ∗: non-specific band).

To assess the efficiency of NSTAT in terms of inducing EBV lytic reactivation in EBVaGC, we determined the expression of various EBV immediately early (*BZLF1*, *BRLF1*), early lytic (*BGLF4*, *BMRF*1) and late (*BLRF2*) lytic genes in NSTAT-treated SNU719 and YCCEL1 cells via qRT-PCR analysis and RNA-sequencing. As shown in Fig. 1A, treatment with 500 nM NSTAT for 24 h significantly induced the transcription of the immediately early lytic gene *BZLF1* in both SNU719 and YCCEL1 cells. NSTAT also reactivated the transcription of the other immediately early lytic gene *BRLF1*, as well as downstream early and late lytic genes (*BGLF4*, *BMRF1* and *BLRF2*) (Fig. 1A). EBV transcriptome profiling demonstrated the induction of multiple EBV lytic genes in NSTAT-treated EBVaGC cells (Fig. 1B). To further determine the minimal NSTAT exposure time required for ZEBRA/BZLF1 induction, we treated EBVaGC cells with 500 nM NSTAT for 1 to 12 h, then removed the drug by washing with fresh medium. The cells were subsequently incubated in this fresh medium before harvesting for protein extraction and *Western blot*ting analysis at 24 h after the initiation of NSTAT treatment. As shown in Fig. 1C, obvious ZEBRA induction was observed in SNU719 and YCCEL1 cells incubated with 500 nM NSTAT for 8 to 10 h.

**Fig. 2:**
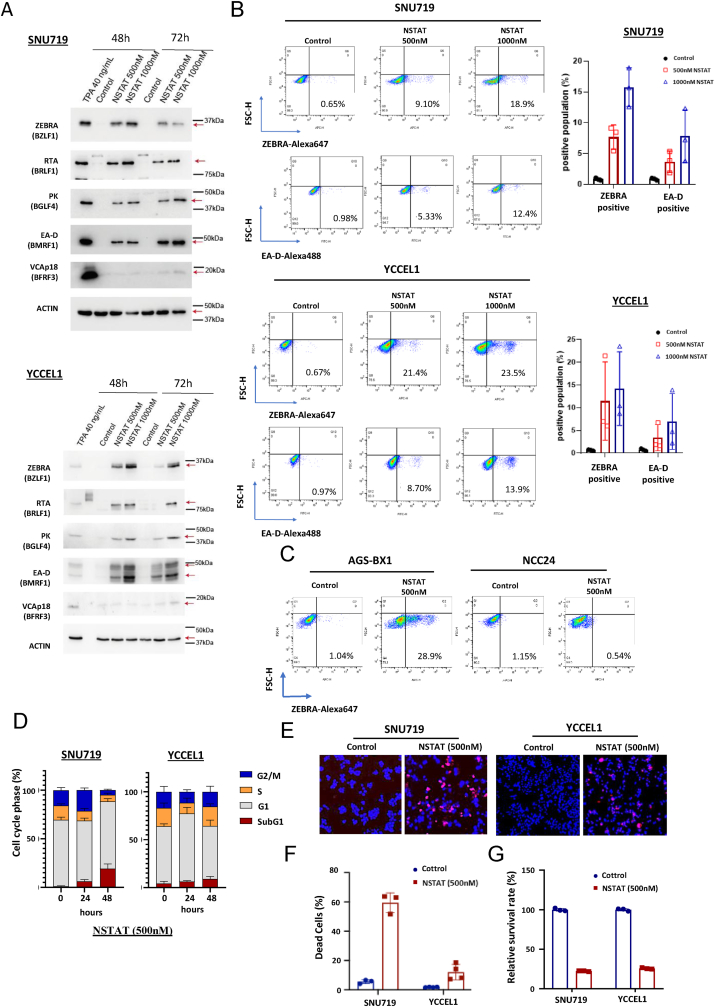
**NSTAT reactivated ZEBRA and downstream EBV lytic proteins in EBVaGC *in vitro.*** (A) Western blotting demonstrated the expression of the EBV lytic proteins ZEBRA, RTA, PK, EA-D and VCAp18 in NSTAT treated SNU719 and YCCEL1 at 48 and 72 h. SNU719 or YCCEL1 cells treated with TPA (40 ng/mL) for 48 h were included as controls. Red arrow: lytic proteins or ACTIN. (B) Flow cytometry analysis revealed the induction of ZEBRA and EA-D expression in NSTAT treated SNU719 and YCCEL1, respectively (*n* = 3 experimental replicates). (C) Cell cycle alterations in NSTAT-treated SNU719 and YCCEL1 at 24 and 48 h (*n* = 3 experimental replicates). Data are presented as the mean ± SD. (D) Live/Dead cell assay indicating the cell viability of SNU719 and YCCEL1 with 48hr treatment of NSTAT. Representative immunofluorence images are shown. (E) Statistical analysis of live/dead cells in NSTAT treated SNU719 and YCCEL1 cells. (F) Inhibition of cell viability was detected in NSTAT-treated SNU719 and YCCLE1 cells using the CCK8 assay.

EBV lytic protein expression was efficiently induced in SNU719 and YCCEL1 cells treated with 500 nM or 1 μM NSTAT. The early lytic proteins PK/BGLF4 and EA-D/BMRF1 were detected in EBVaGC cells treated with NSTAT for 48 and 72 h (Fig. 2A). Flow cytometry analysis demonstrated that approximately 19% and 24% of SNU719 and YCCLE1 cells were ZEBRA-positive after treatment with 1 μM NSTAT treatment for 24 h. Notably, approximately 12%-14% of NSTAT-treated EBVaGC cells were EA-D-positive (Fig. 2B). NSTAT treatment also effectively induced ZEBRA expression in approximately 29% of EBV-reinfected AGS-BX cells (Fig. 2C). However, we found no detectable ZEBRA induction in NSTAT-treated NCC24 cells, which we attribute to the loss of the EBV genome in majority of the cells. The effects of NSTAT on cell cycle regulation and survival in SNU719 and YCCEL1 cells were determined by using flow cytometry, Live/Dead cell viability and CCK8 cell proliferation assays (Fig. 2D–E). As shown in Fig. 2D, obvious increase in the subG 1 populations were observed in both SNU719 and YCCEL1 cells after NSTAT treatment (500 nM) for 48 h. We also found that NSTAT treatment for 24 h led to S phase inhibition in both cell lines. Cytotoxic effect was demonstrated by significant increases in dead cells in both EBVaGC cell lines after treatment with 500 nM NSTAT for 24 h (Fig. 2E). Approximately 60% and 20% were observed as dead cells in NSTAT-treated SNU719 and YCCEL1, respectively (Fig. 2F). Furthermore, dramatic growth inhibition (>60%) was observed in NSTAT-treated EBVaGC cell lines subjected to a CCK-8 cell viability assay (Fig. 2G).

### 3.2 NSTAT induces genome-wide histone acetylation and suppresses c-MYC expression in EBVaGC

**Fig. 3:**
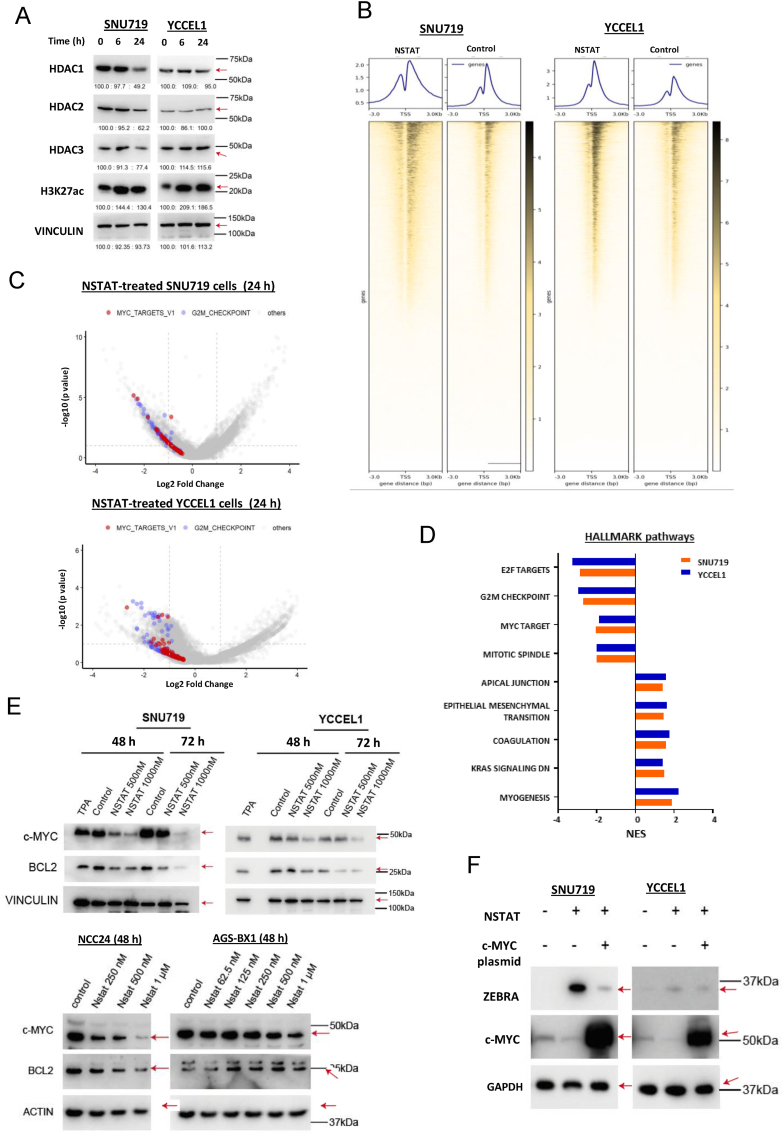
**NSTAT increased genome-wide H3K27 acetylation and suppressed c-MYC expression in EBVaGC *in vitro*.** (A) *Western blot*ting revealed the expression of class I HDACs and H3K27ac in EBVaGC cells. Red arrow: HDAC1, HDAC2, HDAC3, H3K27ac or VINCULIN. (B) ChIP-seq read-density heat maps for H3K27Ac across sequences corresponding to ± 3000bp around TSS sites in SNU719 and YCCEL1 cells after control or NSTAT treatment for 24 h. (C) Volcano plot of RNA-seq data from SNU719 and YCCEL1 cells after control or NSTAT treatment for 24 h. (D) GSEA of differentially expressed genes in SNU719 and YCCEL1 treated with NSTAT for 24 h. Differentially expressed gene sets identified in NSTAT-treated vs. control-treated cells were subjected to “HALLMARK” gene sets. An NES score <0 indicates downregulation after NSTAT treatment. (E) Western blottin*g* detected c-MYC and BCL2 expression in NSTAT-treated SNU719 and YCCEL1 cells at 48 and 72 h. Arrow: c-MYC, BCL2, VINCULIN, or ACTIN. (F) Western blotting detected ZEBRA expression in NSTAT-treated SNU719 and YCCEL1 cells exhibiting ectopic expression of c-MYC at 48 h. Arrow: ZEBRA, c-MYC or GAPDH.

In addition to the effects on EBV lytic induction, we also evaluated the HDAC inhibitor activity of NSTAT in the two EBVaGC cell lines using *Western blot*ting, ChIP-sequencing and total RNA sequencing. We observed an obvious increase in H3K27ac expression in the EBVaGC cells treated with NSTAT for 6 and 24 h (Fig. 3A). We then conducted H3K27ac ChIP-seq to investigate whether NSTAT treatment would alter the H3K27ac landscape of the SNU719 and YCCLE1 genomes. Consistent with the increase in H3K27ac, we found that NSTAT treatment efficiently activated genome-wide H3K27ac signals located 3000 bp upstream to 3000 bp downstream of the annotated transcription start sites (TSSs) (Fig. 3B). NSTAT treatment also induced obvious transcriptional changes, and 3045 and 1697 DEGs were identified in NSTAT-treated SNU719 and YCCEL1 cells, respectively (Fig. 3C). Through GSEA analysis with HALLMARK pathways, we revealed that NSTAT treatment suppressed the targets of MYC and pathways of cell cycle regulations, such as the E2F targets, G2M checkpoint and mitotic spindle pathways (Fig. 3D). The downregulated DEGs involved in the G2M checkpoint and MYC target pathways were also shown in the volcano plots of RNA-seq analysis (Fig. 3C). This finding is concordant with the increase in the G1 and G2M subpopulations of SNU719 and YCCEL1 cells after NSTAT treatment (Fig. 2D). We performed Western blotting to confirm that c-MYC and BCL2 expression was obviously reduced in SNU719 YCCEL1, AGS-EBV and NCC24 cells after NSTAT treatment (Fig. 3E). Downregulation of the anti-apoptotic protein BCL2 was also detected in these NSTAT-treated cell lines. NSTAT-induced ZEBRA expression was suppressed in EBVaGC cells exhibiting ectopic expression of c-MYC (Fig. 3F). These findings indicate that NSTAT-mediated c-MYC suppression contributes to EBV lytic reactivation and growth inhibition in EBVaGC cells [28].

### 3.3 NSTAT induces EBV lytic genes and represses c-MYC in EBVaGC in vivo

**Fig. 4:**
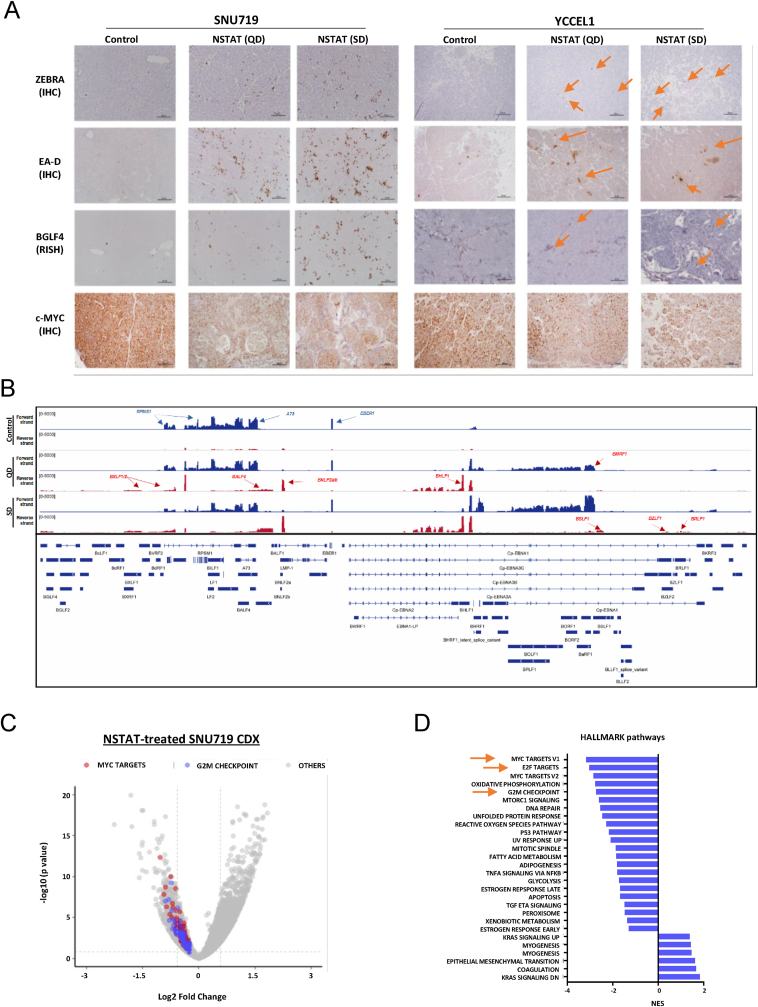
**NSTAT reactivated the expression of EBV lytic genes and suppressed c-MYC expression in EBVaGC CDXs.** (A) Using immunohistochemical staining, changes in the expression of ZEBRA, EA-D and c-MYC were detected in the representative sections of tumor from NOD SCID mouse models at 6 days after orally adminstered NSTAT (QD or SD) in SNU719 models and at 3 days in YCCEL1 models (QD or SD). Expression of EBV lytic gene *BGLF4* was also detected using RNAscope RNA *in situ* hybridization (RISH). Scale bar = 50 μm. Arrows: ZEBRA or *BGLF4* signals. (B) Representative EBV transcriptome profiles from SNU719 tumors in NOD SCID mouse models after short-term oral administration of NSTAT (QD or SD). EBV lytic and latent genes are indicated by red and blue arrows respectively. (C) Differentially expressed genes identified in tumors from SNU719 NOD-SCID mouse models at 6 days after short-term treatment with NSTAT versus control (0.001% HCl; n = 2). (D) GSEA of differentially expressed genes in the SNU719 mouse model after short-term NSTAT treatment. Differentially expressed gene sets identified in the tumor tissues of NSTAT-treated vs. control-treated mice were subjected to “HALLMARK” gene sets. An NES score <0 indicates downregulation after NSTAT treatment.

We examined the ability of NSTAT to induce EBV lytic reactivation *in vivo* in the SNU719 and YCCEL1 CDX mouse models. In NOD SCID mice subjected to short-term NSTAT treatment (2-4 days, once a day (QD) or split dosing with twice a day (SD)), obvious induction of EBV lytic gene products, namely ZEBRA, EA-D and *BGLF4* transcripts, was detected in the SNU719 and YCCEL1 tumors by immunohistochemistry (IHC) and RNAscope RNA-*in situ* hybridization (RISH) (Fig. 4A). Reduced c-MYC expression was also observed in NSTAT-treated SNU719 and YCCEL1 tumors (Fig. 4A). The induction of multiple EBV immediate-early (e.g., *BZLF1*) and early (e.g., *BSLF1*, *BXLF1/2*) lytic genes in NSTAT-treated SNU719 tumors was also demonstrated by RNA sequencing analysis (Fig. 4B). Compared with QD treatment, SD treatment with NSTAT more potently induced EBV lytic gene expression in SNU719 cells. *In vivo* short-term NSTAT treatment also led to obvious changes in the cellular transcription profile, and a total of 296 DEGs were identified (Fig. 4C). Consistent with the *in vitro* study, GSEA revealed the inhibition of MYC targets and cell cycle regulation pathways (e.g., E2F targets, G2M checkpoint, mitotic spindle) (Fig. 4D). Repression of the MTORC1 signaling, fatty acid metabolism and TNFA/NFKB signaling pathways was identified in our short-term *in vivo* study. These findings confirm the *in vivo* effects of NSTAT in terms of reactivation of the EBV lytic cycle and inhibition of the oncogenic properties of EBVaGC.

### 3.4 *In vivo* therapeutic efficacy of NSTAT in EBVaGC

**Fig. 5:**
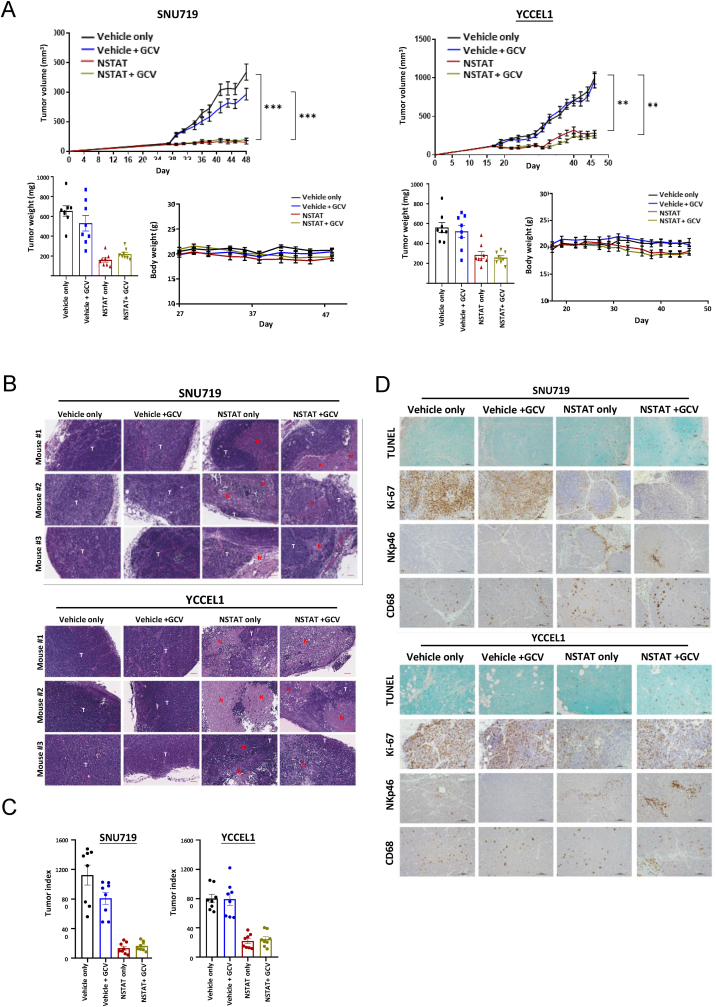
***In vivo* inhibition of EBVaGC by NSTAT-based lytic induction treatment.** The average tumor volumes, individual tumor volumes and body weights were measured throughout treatment in (A) SNU719 and YCCEL1 CDXs (*n* = 8 mice/group); data are presented as the mean ± SEM. Student's t-test was used to calculate p-values. (B) Representative images of hematoxylin and eosin stained FFPE sections harvested from NSTAT-treated SNU719 and YCCEL1 CDXs. (C) The tumor index of each harvested EBV-positive tumor was determined after treatment with NSTAT alone, combined NSTAT and GCV, GCV alone and vehicle controls. Tumor index = tumor volume × percentage of the hematoxylin-positive area. Significant tumor growth inhibition was observed in both SNU719 and YCCEL1 xenografts treated with NSTAT alone or combined with GCV (*n* = 8 mice/group). Data are presented as the mean ± SD. (D) TUNEL assay illustrating the preferential killing effect of NSTAT with GCV in the tumors of NOD SCID mice implanted with SNU719 and YCCEL1 CDXs. Expression of Ki-67, NKp46 and CD68 in the tumors was detected by IHC assay

To evaluate the anti-tumor efficacy of NSTAT against EBVaGC *in vivo*, NOD SCID mice harboring SNU719 and YCCEL1 CDXs were orally administered 10 mg/kg NSTAT or vehicle control in the presence or absence of 30 mg/kg GCV daily, beginning when the tumor volume reached around approximately 80-100 mm^3^. As shown in Fig. 5A and Supplementary Fig. S4, NSTAT alone or in combination with GCV significantly inhibited tumor growth in both the SNU719 and YCCEL1 xenografts when compared with the vehicle control. In EBVaGC treated with NSTAT alone or in combination with GCV, we also observed massive necrotic lesions and an increase in apoptotic cells in the harvested residual tumors (Fig. 5B–D). Ki-67 cell proliferation signals were reduced in the NSTAT-treated tumors relative to those treated with vehicles or GCV only (Fig. 5D). Furthermore, we observed the accumulation and infiltration of natural killer (NK) cells and macrophages in either residual tumors or adjacent necrotic regions in mice treated with NSTAT alone or in combination with GCV (Fig. 5D). In addition to the absence of changes in body weight, no tissue damage was observed in multiple organs of the mice in both control and treatment groups (Fig. 5A and Supplementary Fig. S5). Although treatment with NSTAT alone and in combination with GCV exerted similarly potent growth inhibitory effects, our *in vitro* study demonstrated that the combination of GCV and NSTAT treatment significantly reduced the production of infectious EBV virions by EBVaGC cells. The generation of EBV-reinfected Akata cells following incubation with supernatants from NSTAT-treated SNU719 and YCCLE1 cells was reduced when NSTAT was combined with GCV (Supplementary Fig. S6). These findings demonstrate the potent antitumor effects and safety of combined NSTAT and GCV treatment in preclinical models of EBVaGC.

## 4 Discussion

Over the three decades, researchers have identified several chemical activators that trigger the viral latency-lytic switch to induce lytic reactivation in EBV-infected cells [7,8]. Among these, HDAC inhibitors appear to efficiently reactivate EBV lytic genes in multiple EBV-positive lymphoma and epithelial cancer cells. Nevertheless, few of these inhibitors have been implemented into the clinical management of EBV-associated cancers due to the broad spectra of cytotoxicity and cell-context specific activity in EBV lytic reactivation. While our recent study on a synthetic *BZLF1*-targeted transcriptional activator mRNA demonstrated the potent antitumor effects of EBV lytic induction therapy against EBV-positive epithelial cancers, clinically available chemical inducers for the efficient treatment of EBVaGC remain lacking [15]. Although the clinically approved HDAC inhibitors, SAHA and romidepsin can induce BZLF1 expression in the EBV-reinfected GC cell line AGS-BX1, we found that these inhibitors failed to induce lytic reactivation in two native EBVaGC cancer cell lines, SNU719 and YCCEL1 [28]. This difference between the responses of native EBVaGC and EBV-reinfected GC cell lines to lytic reactivation can be attributed to their specific genetic and epigenetic profiles. During EBVaGC tumorigenesis, various subtype-specific genetic and epigenetic changes are through to be acquired to maintain persistent EBV latency. These restriction factors may be absence in AGC cells, which are derived from a gastric cancer of the chromosomally unstable (CIN) molecular subtype.

NSTAT is a potent class I-selective HDAC inhibitor with good oral bioavailability and has been shown to induce apoptosis in myeloma cells at nanomolar concentrations. A phase 1/2 clinical trial of combined NSTAT and VGCV therapy demonstrated efficacy and safety in patients with EBV-positive lymphoid malignancies. The treatment was generally well tolerated by patients, although 31-38% reported adverse events such as nausea, thrombocytopenia, neutropenia, anemia and constipation [16]. In this study, we further demonstrated the therapeutic potential of this lytic induction therapy in the representative patient-derived EBVaGC preclinical models SNU719 and YCCEL1. Importantly, we revealed that NSTAT not only induces EBV lytic gene expression, but also targets c-MYC and cell cycle regulation pathways in EBVaGC both *in vitro* and *in vivo.* NSTAT-mediated BCL2 and c-MYC repression may promoting apoptosis and cell cycle arrest in EBVaGC, as previously reported in myeloma cells [29]. Dysregulation of c-MYC expression plays an important role in maintaining latency in EBV-infected cells. A recent study revealed a c-MYC linked transcriptional network required to suppress EBV lytic cycle. Depletion of c-MYC alters the three-dimensional genomic architecture of EBV by looping viral genomic elements to *BZLF1* promoter and subsequently reactivating EBV lytic replication [27]. We also recently demonstrated that c-MYC knock-down led to the induction of the EBV immediately early lytic protein in multiple EBV-positive NPC cell lines [30]. These findings suggest that c-MYC is key mechanism targeted during NSTAT-induced EBV lytic reactivation in EBVaGC. Notably, NSTAT had a limited suppressive effect on c-MYC expression in AGS-BX1 cells, which were not derived from an EBVaGC molecular subtype tumor (Fig. 3E). We also found that other HDAC inhibitors including SAHA, romidepsin and NaB repressed c-MYC expression in both SNU719 and YCCEL1 cells (Supplementary Fig. S7). This finding indicates that c-MYC downregulation is a common effect of HDAC inhibitors on EBVaGC cells. As a potent antitumor drug against EBVaGC, the mechanisms of action of NSTAT include targeting oncogenic pathways, promoting apoptosis and inducing EBV lytic reactivation. Our *in vivo* study revealed no obvious GCV-mediated bystander killing effect when the growth inhibitory effects were compared between the groups with NSTAT alone or in combination with GCV. This is likely to be attributable the potent cytolytic effect of NSTAT on EBVaGC tumor cells or the low release efficiency of cytotoxic GCV to adjacent tumor cells in our CDX mouse models. A similar observation was reported in a study of combined valproic acid, gemcitabine and GCV treatment in EBV-positive NPC xenograft models [31]. The potential synergistic effects of GCV against EBVaGC cells treated with various doses of NSTAT were investigated in a SynergyFind 3.0 analysis [32]. As shown in Supplementary Fig. S8, only a weak synergistic or additive effect was observed in SNU719 and YCCEL1 cells treated with a combination of low-dose (<500 nM) NSTAT and various doses of GCV. Both the *in vitro* and *in vivo* studies revealed a limited bystander effect of combined NSTAT and GCV treatment in preclinical EBVaGC models. Although NSTAT alone exerted a potent growth inhibitory effect against EBVaGC, combination treatment with GCV is essential for inhibiting viral DNA replication and preventing the dissemination of infective EBV virions during lytic reactivation. In our *in vitro* study, we demonstrated that GCV reduced the production of infectious EBV virions in SNU719 and YCCLE1 cells subjected to combination treatment (Supplementary Fig. S6). The findings confirm the indispensable role of GCV in lytic induction therapy for EBV-associated cancer.

Our study is limited by the lack of available EBVaGC immunocompetent mouse models for investigating host immune response against the induced EBV lytic antigens. Nevertheless, we found NSTAT alone or in combination with GCV led to accumulation and infiltration of NK cells and macrophages in the tumors of EBVaGC NOD SCID mouse models. Our preliminary finding suggests that the expression of EBV lytic proteins may trigger the host's innate immune responses to EBVaGC after NSTAT treatment. As NOD SCID mice lack B and T lymphocytes, the establishment of humanized mouse models of EBVaGC would allow us to accurately elucidate both innate and adaptive immune responses to the EBV lytic antigens induced by NSTAT treatment. A recent study reported the high potency of EBV-specific T cells against both lytic and latent antigens [33]. While NSTAT treatment is believed to enhance the immunogenicity of EBVaGC, the combination of NSTAT with immune checkpoint inhibitors, such as PD1/PDL1 inhibitors, is anticipated as an efficient therapeutic strategy for eradicating this EBV-associated epithelial cancer.

To address the emerging need for targeted therapies for EBVaGC, we have demonstrated the antitumor efficacy and safety profile of combined NSTAT and GCV treatment in EBVaGC preclinical models. Strikingly, our findings provide new insights into the unique action of NSTAT against EBVaGC, a specific subtype of gastric cancer. NSTAT-mediated c-MYC repression contributes to the induction of EBV lytic reactivation and the inhibition of cell proliferation and survival in EBVaGC. This preclinical study thus provides important evidence supporting a further evaluation of the therapeutic efficacy of NSTAT treatment for EBVaGC in future clinical trials.

## CRediT authorship contribution statement

**Man Wu:** Writing – original draft, Validation, Software, Project administration, Methodology, Investigation, Formal analysis, Data curation. **Shin Yee Hui:** Writing – original draft, Validation, Methodology, Investigation, Formal analysis, Data curation. **Andrew Skora:** Project administration, Methodology, Investigation, Formal analysis, Data curation. **Yuk Yu Chan:** Methodology, Investigation, Formal analysis, Data curation. **Grace Tin-Yun Chung:** Methodology, Investigation, Formal analysis. **Lili Li:** Resources, Methodology, Investigation, Formal analysis. **Qian Tao:** Resources, Methodology, Investigation, Formal analysis. **Dajiang Guo:** Methodology, Investigation, Formal analysis. **Christopher W. Dawson:** Methodology, Investigation, Formal analysis. **Lawrence S. Young:** Writing – review & editing. **Chi Man Tsang:** Writing – review & editing, Methodology, Investigation, Formal analysis, Data curation. **Ayman El-Guindy:** Writing – review & editing, Validation, Supervision, Project administration, Funding acquisition, Conceptualization. **Kwok Wai Lo:** Writing – review & editing, Writing – original draft, Validation, Supervision, Resources, Project administration, Investigation, Funding acquisition, Formal analysis, Data curation, Conceptualization.

## Funding

K.W.L. was supported by the Innovation and Technology Fund (Midstream Research Programme for Universities - MRP/036/21X); Research Grant 10.13039/100024337Council, Hong Kong (Areas of Excellence Scheme – AoE/M-401/20; General Research Fund − 14101721), 10.13039/100018696Health and Medical Research Fund (08191046). C.M.T. was supported by the General Research Fund (14124925, 14113620, 14114523, 17122420), Early Career Scheme (24114922), 10.13039/100018696Health and Medical Research Fund (09203176), and Faculty Innovation Award (FIA2020/A/01) of the 10.13039/501100004853Chinese University of Hong Kong.

## Declaration of competing interest

Man Wu, Chi Man Tsang and Kwok-Wai Lo are cofounders of ACE NanoMed Ltd. Kwok-Wai Lo is a member of editorial board of the Tumor Virus Research journal and received research funding from Viracta Therapeutics. Ayman El-Guindy and Andrew Skora were employees for Viracta Therapeutics.

## Data Availability

RNA-seq data are available from The European Nucleotide Archive (ENA) database: PRJEB101317.

## References

[1] Tsao S.W. (2015). The role of epstein-barr virus in epithelial malignancies. J. Pathol..

[2] Yang J. (2020). Epstein-barr virus-associated gastric cancer: a distinct subtype. Cancer Lett..

[3] Salnikov M.Y., MacNeil K.M., Mymryk J.S. (2024). The viral etiology of EBV-Associated gastric cancers contributes to their unique pathology, clinical outcomes, treatment responses and immune landscape. Front. Immunol..

[4] Norwood D.A. (2022). Gastric cancer: emerging trends in prevention, diagnosis, and treatment. Gastroenterol. Clin. N. Am..

[5] Sundar R. (2025). Gastric cancer. Lancet.

[6] Soldan S.S., Messick T.E., Lieberman P.M. (2022). Therapeutic approaches to epstein-barr virus cancers. Curr. Opin. Virol..

[7] Hau P.M. (2020). Targeting epstein-barr virus in nasopharyngeal carcinoma. Front. Oncol..

[8] Yiu S.P.T. (2020). Lytic induction therapy against epstein-barr virus-associated malignancies: past, present, and future. Cancers (Basel).

[9] Perrine S.P. (2007). A phase 1/2 trial of arginine butyrate and ganciclovir in patients with epstein-barr virus-associated lymphoid malignancies. Blood.

[10] Wildeman M.A. (2012). Cytolytic virus activation therapy for epstein-barr virus-driven tumors. Clin. Cancer Res..

[11] Hui E.P. (2013). Phase I trial of recombinant modified vaccinia Ankara encoding epstein-barr viral tumor antigens in nasopharyngeal carcinoma patients. Cancer Res..

[12] Colevas A.D. (2025). First-in-human clinical trial of a small-molecule EBNA1 inhibitor, VK-2019, in patients with epstein-barr-positive nasopharyngeal cancer, with pharmacokinetic and pharmacodynamic studies. Clin. Cancer Res..

[13] Messick T.E. (2019). Structure-based design of small-molecule inhibitors of EBNA1 DNA binding blocks epstein-barr virus latent infection and tumor growth. Sci. Transl. Med..

[14] Soldan S.S. (2021). EBNA1 inhibitors have potent and selective antitumor activity in xenograft models of epstein-barr virus-associated gastric cancer. Gastric Cancer.

[15] Wu M. (2024). Synthetic BZLF1-targeted transcriptional activator for efficient lytic induction therapy against EBV-Associated epithelial cancers. Nat. Commun..

[16] Haverkos B. (2023). Targeted therapy with nanatinostat and valganciclovir in recurrent EBV-Positive lymphoid malignancies: a phase 1b/2 study. Blood Adv..

[17] Shi M.Q. (2024). Advances in targeting histone deacetylase for treatment of solid tumors. J. Hematol. Oncol..

[18] Li Y., Seto E. (2016). HDACs and HDAC inhibitors in cancer development and therapy. Cold Spring Harb. Perspect. Med..

[19] Park J.G. (1997). Establishment and characterization of human gastric carcinoma cell lines. Int. J. Cancer.

[20] Kim D.N. (2013). Characterization of naturally epstein-barr virus-infected gastric carcinoma cell line YCCEL1. J. Gen. Virol..

[21] Ku J.L. (2012). Establishment and characterization of six human gastric carcinoma cell lines, including one naturally infected with epstein-barr virus. Cell. Oncol..

[22] Choi C.K. (2015). Identification of novel small organic compounds with diverse structures for the induction of epstein-barr virus (EBV) lytic cycle in EBV-Positive epithelial malignancies. PLoS One.

[23] Lin W. (2018). Establishment and characterization of new tumor xenografts and cancer cell lines from EBV-positive nasopharyngeal carcinoma. Nat. Commun..

[24] Love M.I., Huber W., Anders S. (2014). Moderated estimation of fold change and dispersion for RNA-Seq data with DESeq2. Genome Biol..

[25] Liberzon A. (2015). The molecular signatures database (MSigDB) hallmark gene set collection. Cell Syst..

[26] Wu T. (2021). clusterProfiler 4.0: a universal enrichment tool for interpreting omics data. Innovation.

[27] Guo R. (2020). MYC controls the epstein-barr virus lytic switch. Mol. Cell.

[28] Hui K.F., Chiang A.K. (2010). Suberoylanilide hydroxamic acid induces viral lytic cycle in epstein-barr virus-positive epithelial malignancies and mediates enhanced cell death. Int. J. Cancer.

[29] Smith E.M. (2015). The combination of HDAC and aminopeptidase inhibitors is highly synergistic in myeloma and leads to disruption of the NFκB signalling pathway. Oncotarget.

[30] Chan Y.Y. (2023). Abstract 6226: therapeutic vulnerability of MTAP-Deleted nasopharyngeal carcinoma by MAT2A inhibitors. Cancer Res..

[31] Novalić Z. (2017). Cytolytic virus activation therapy and treatment monitoring for epstein-barr virus associated nasopharyngeal carcinoma in a mouse tumor model. J. Med. Virol..

[32] Ianevski A. (2022). SynergyFinder 3.0: an interactive analysis and consensus interpretation of multi-drug synergies across multiple samples. Nucleic Acids Res..

[33] Sharma S. (2024). Cotargeting EBV lytic as well as latent cycle antigens increases T-cell potency against lymphoma. Blood Adv..

