# Supplementary material for: Targeting EBV-associated gastric cancer by lytic induction therapy with nanatinostat
Source: Tumour Virus Res. 2026 Jun 27;22:200346. doi: 10.1016/j.tvr.2026.200346 (PMC13332451; doi:10.1016/j.tvr.2026.200346)
Supplement: Multimedia component 1 [file mmc1.pdf]

Supplement Figure 1

NSTAT

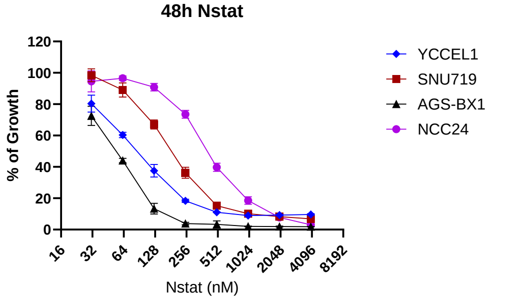

Cell Line	IC_{50} (nM)
SNU719	190.60
YCCEL1	87.28
NCC24	421.70
AGS-BX1	52.92

SAHA

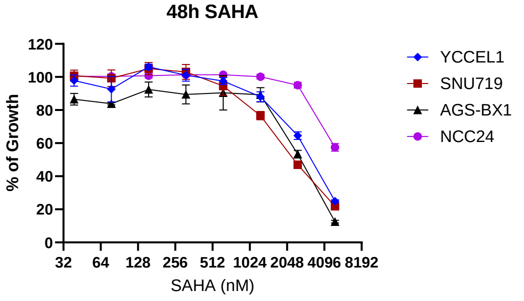

Cell Line	IC_{50} (nM)
SNU719	2409
YCCEL1	3163
NCC24	5401
AGS-BX1	2588

NaB

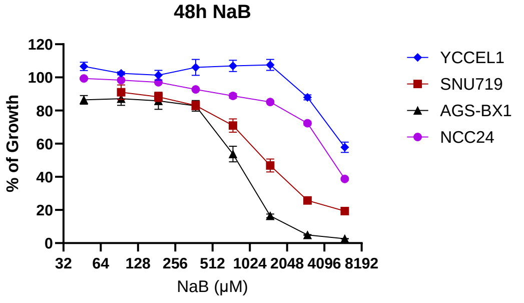

Cell Line	IC_{50} (μM)
SNU719	1402
YCCEL1	6630
NCC24	4867
AGS-BX1	747.6

TSA

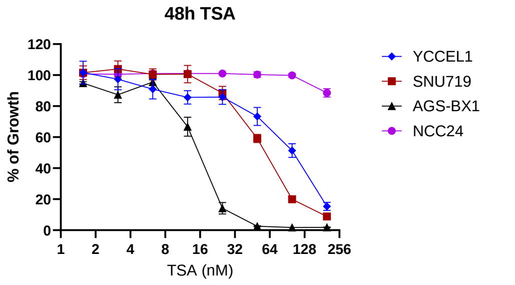

Cell Line	IC_{50} (nM)
SNU719	58.32
YCCEL1	88.90
NCC24	NA
AGS-BX1	15.16

Romidepsin

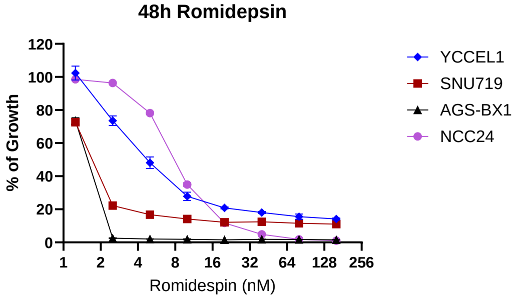

Cell Line	IC_{50} (nM)
SNU719	1.75
YCCEL1	6.03
NCC24	8.11
AGS-BX1	1.46

Supplementary Figure 1: Dose-response analysis in EBV-positive GC cell lines treated with HDAC inhibitors for 48 h. Forty-eight-hour dose-response analysis of NSTAT and other HDAC inhibitors was performed in three EBVaGC cell lines SNU719, YCCEL1 and NCC24 and an EBV-reinfected G. The IC_{50} values of HDAC inhibitors in EBV-positive GC cell lines are indicated.

Supplement Figure 2

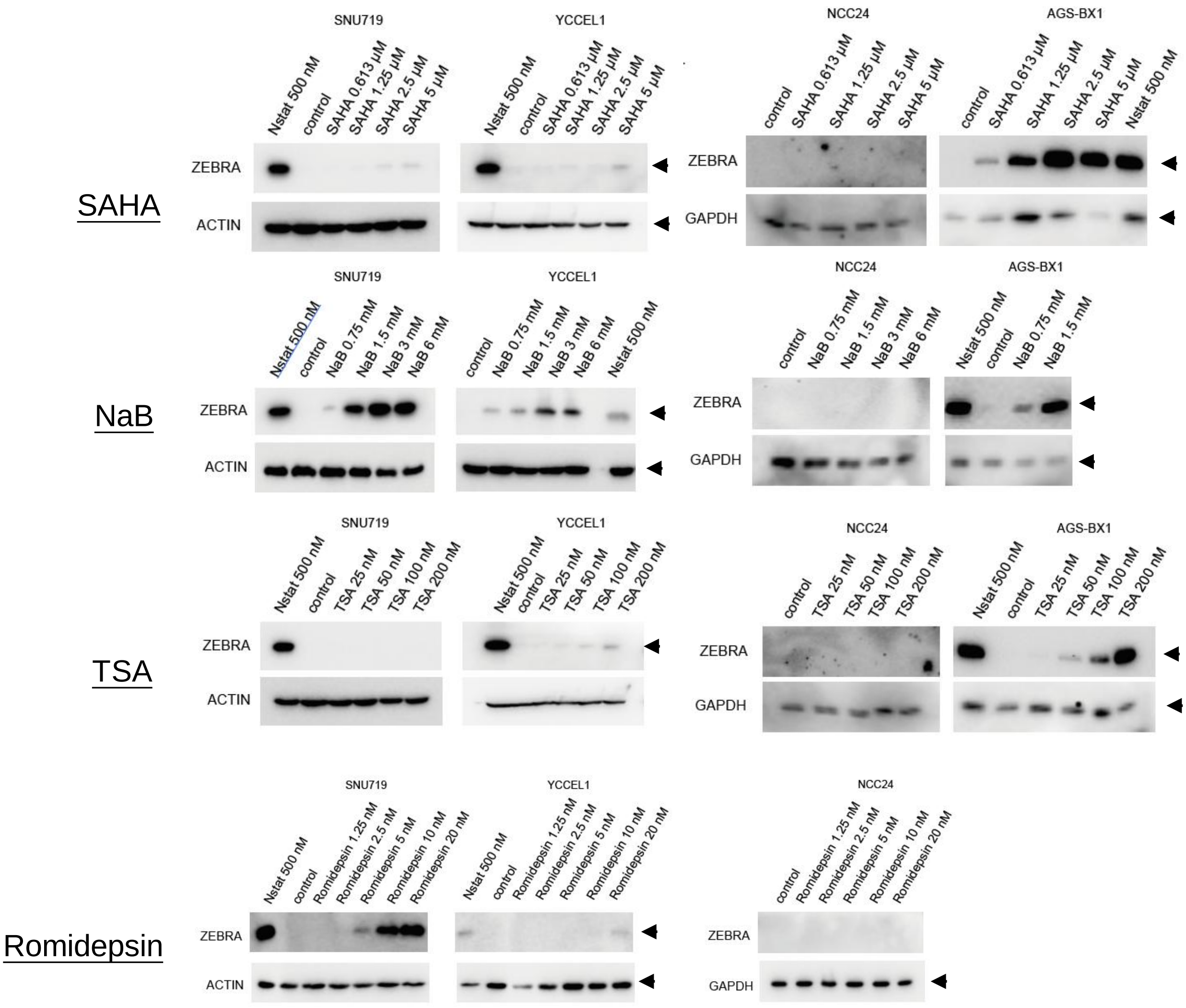

Supplementary Figure 2: Western blotting was used to examine ZEBRA expression in EBV-positive GC cell lines after 48-h treatment with the HAC inhibitors including NSTAT, SAHA, NaB, TSA, Romidepsin. .

Supplement Figure S3

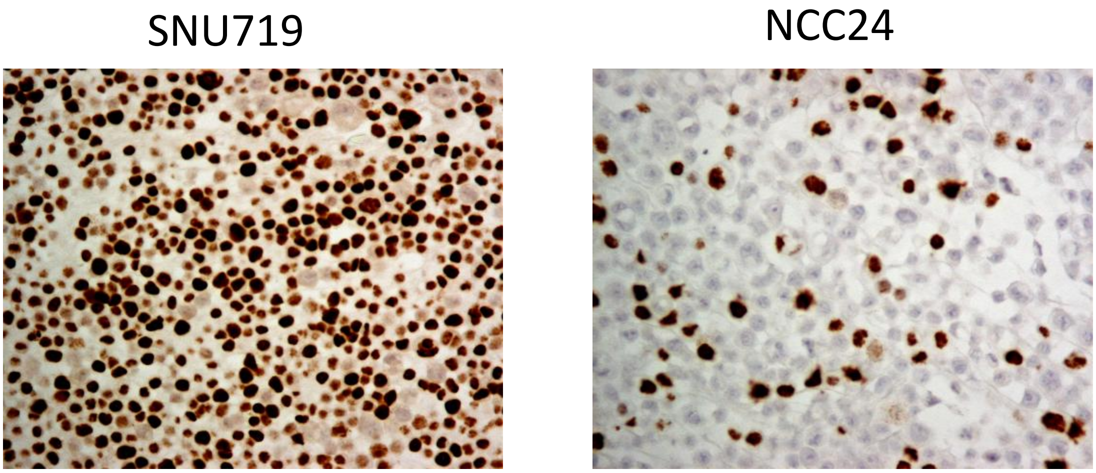

Supplement Figure 3: RNAscope *in situ* hybridization analysis was used to examine *EBER1* expression in EBVaGC cell lines SNU719 and NCC24. *EBER1* transcripts was detected in only 10-20% of NCC24 cells.

Supplement Figure S4

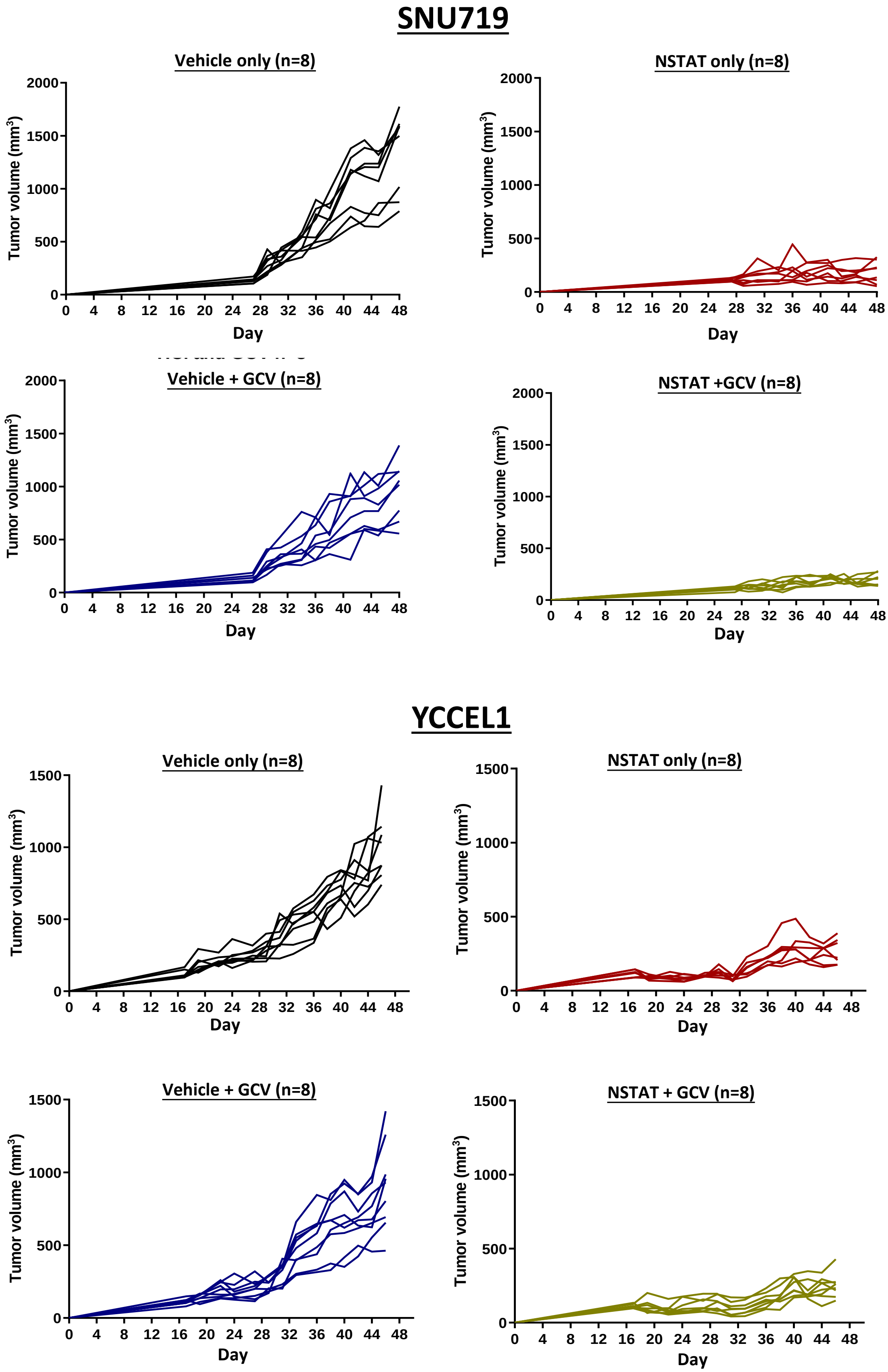

Supplementary Figure S4: IndividualSNU719 and YCCEL1 CDX tumor volumes measured throughout the treatment periods.

Supplement Figure S5

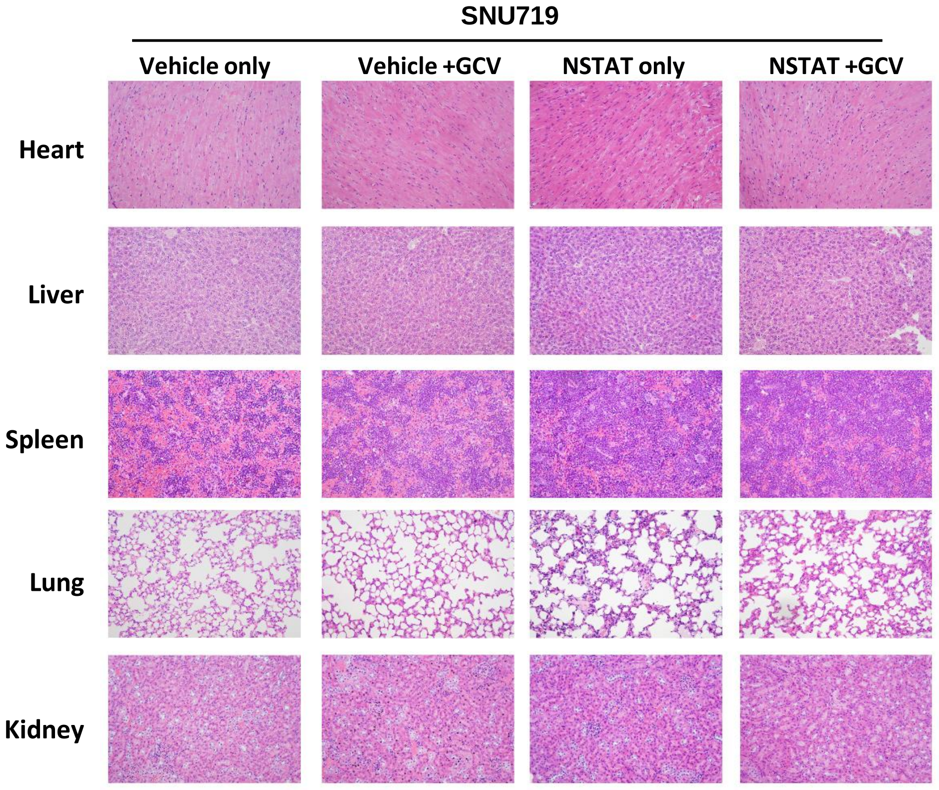

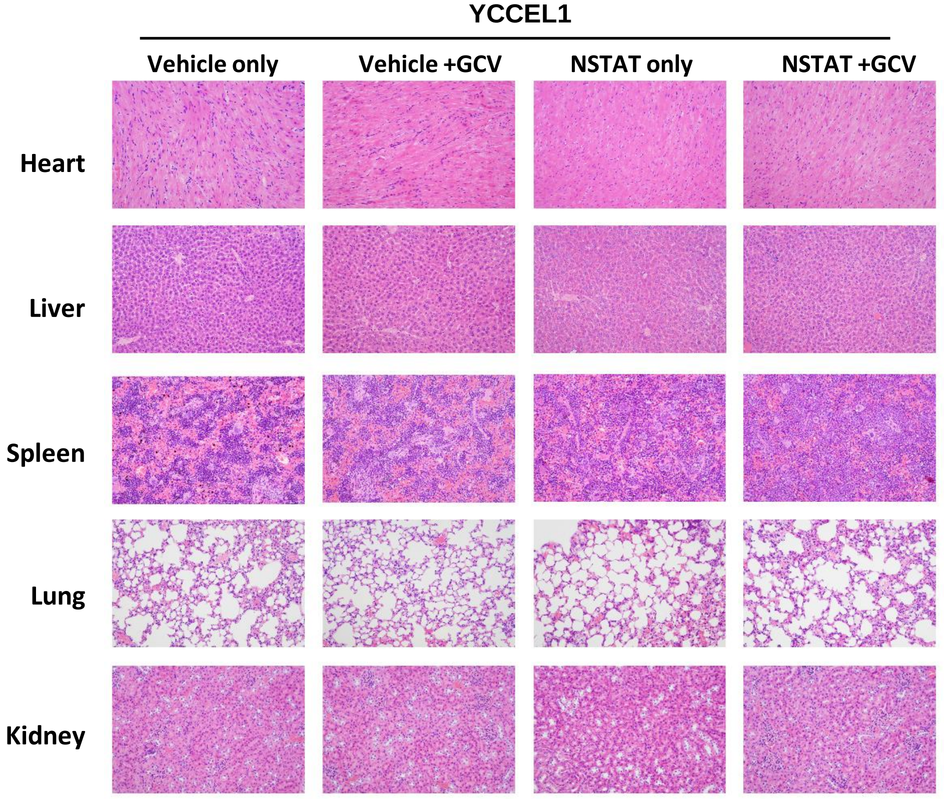

Supplementary Figure S5: Effects of NSTAT on tumor tissues and normal organs. Representative images depict hematoxylin and eosin stained FFPE tissue sections harvested from NSTAT-treated SNU719 and YCCEL1 CDXs.

.

Supplement Figure S6

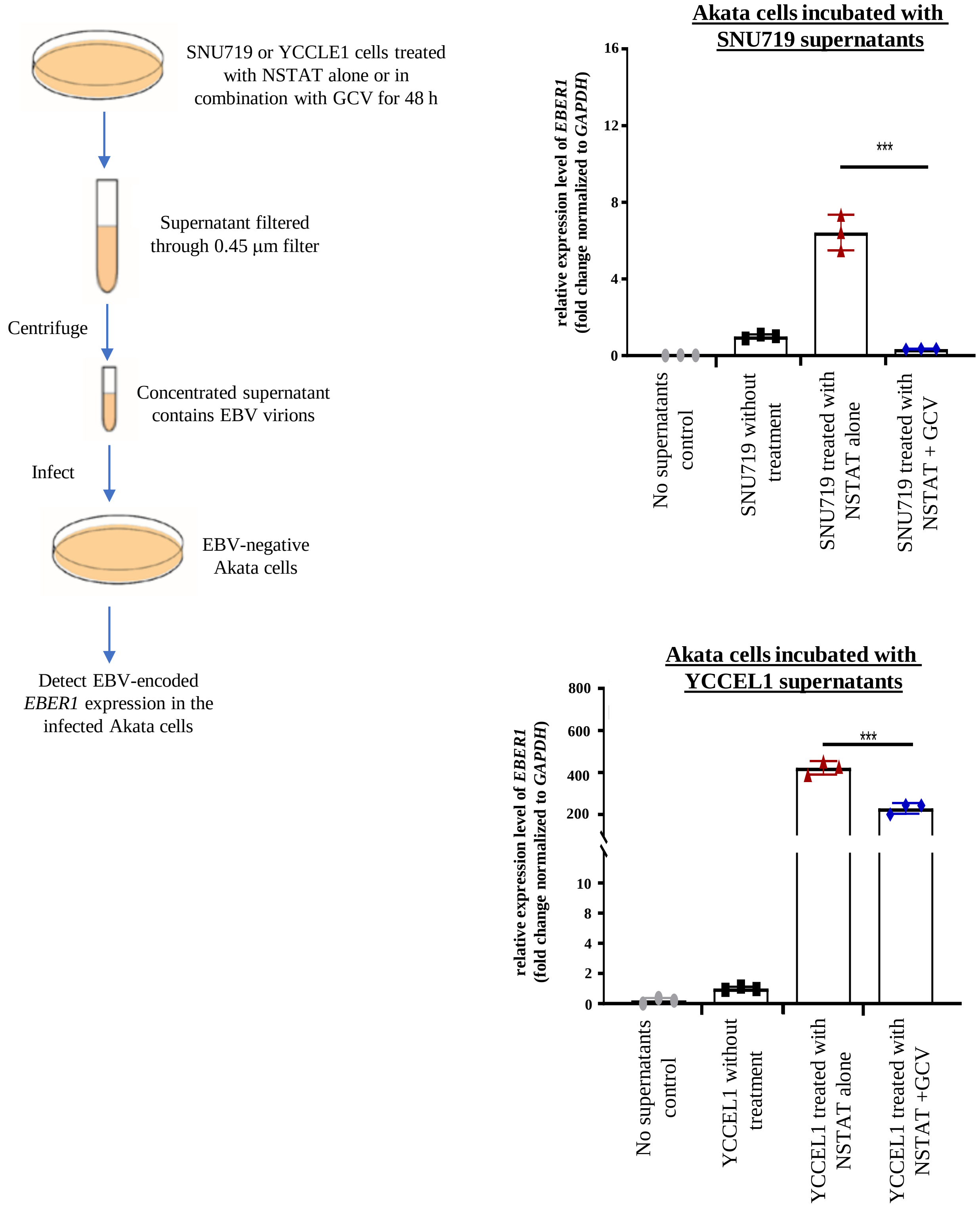

Supplementary Figure S6: The combination of GCV and NSTAT treatment reduced the production of infectious EBV virions by EBVaGC cells. Production of infections EBV infectious EBV virions was detected in SNU719 and YCCEL1 cells treated with NSTAT alone or in combination with GCV for 48 h. Quantitative RT-PCR was used to detect the expression levels of *EBER1* transcripts was detected in the EBV-negative Akata cells incubated with supernatants collected from the SNU719 or YCCEL1 cells treated with NSTAT alone or in combination with GCV. Data are presented as mean ± SD.

Supplement Figure S7

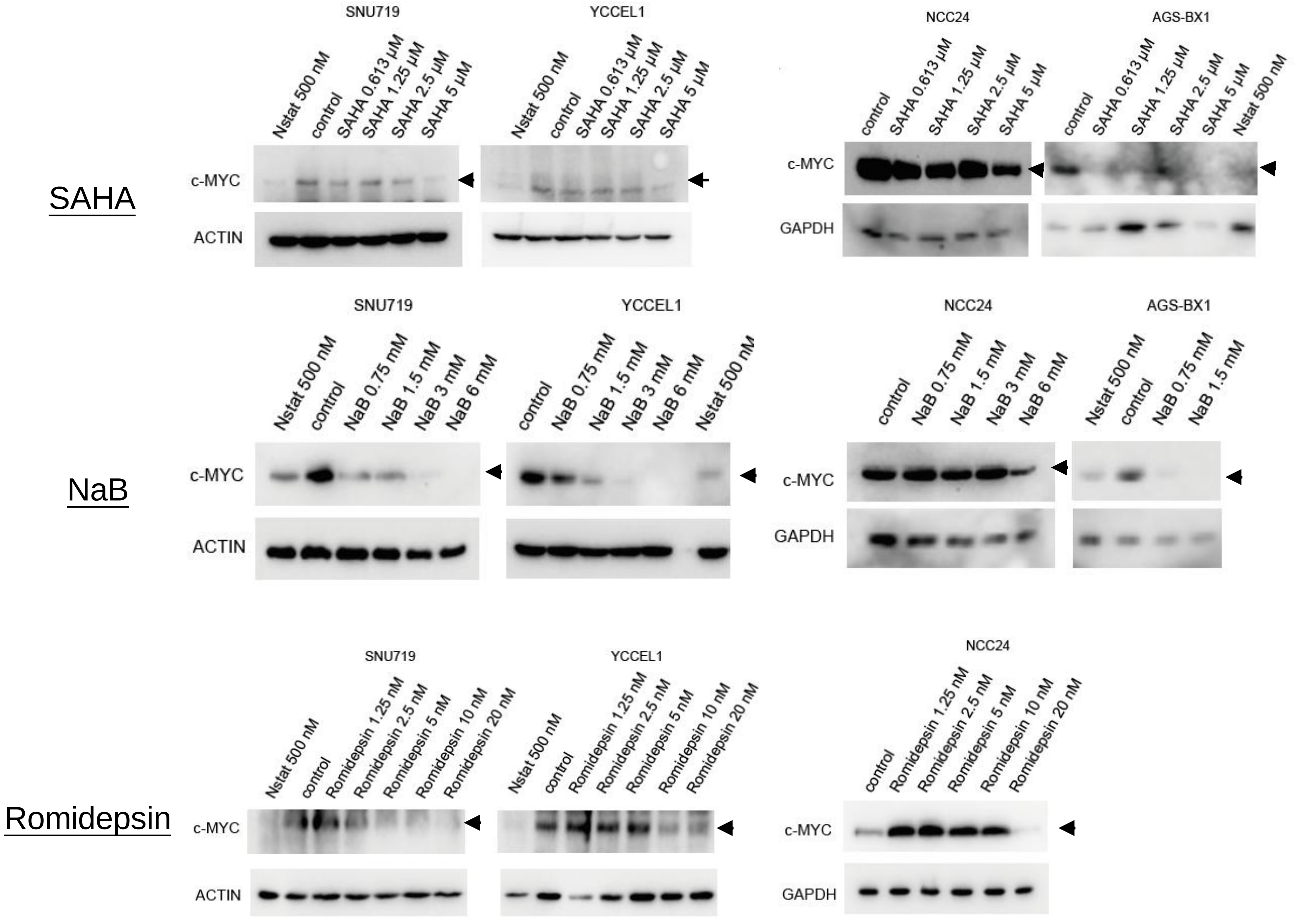

Supplementary Figure S7: Western blotting was used to examine c-MYC expression in EBV-positive GC cell lines after 48-h treatment with the HAC inhibitors including SAHA, NaB and Romidepsin.

Supplement Figure S8

SNU719

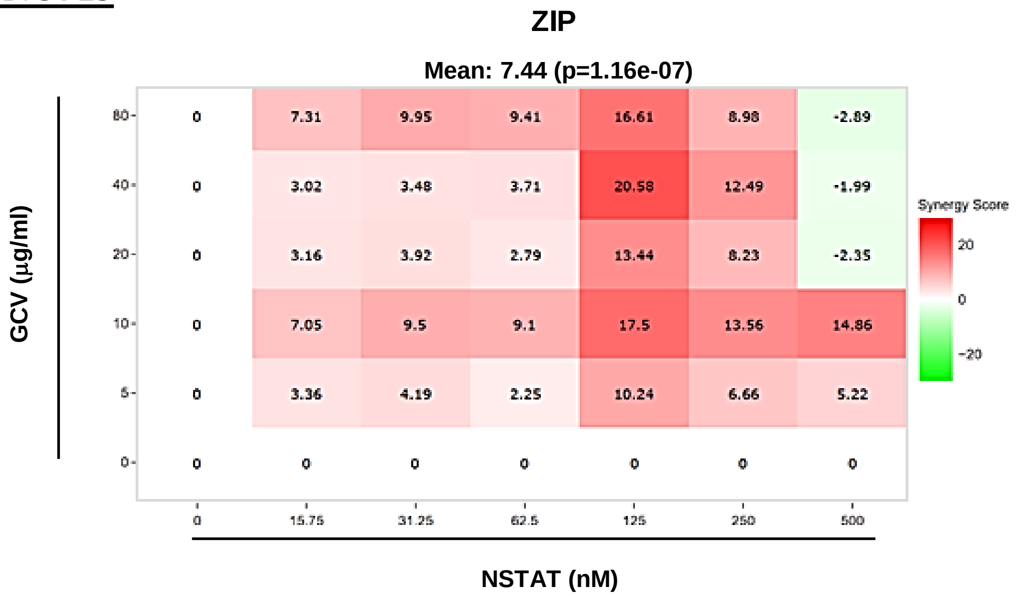

YCCEL1

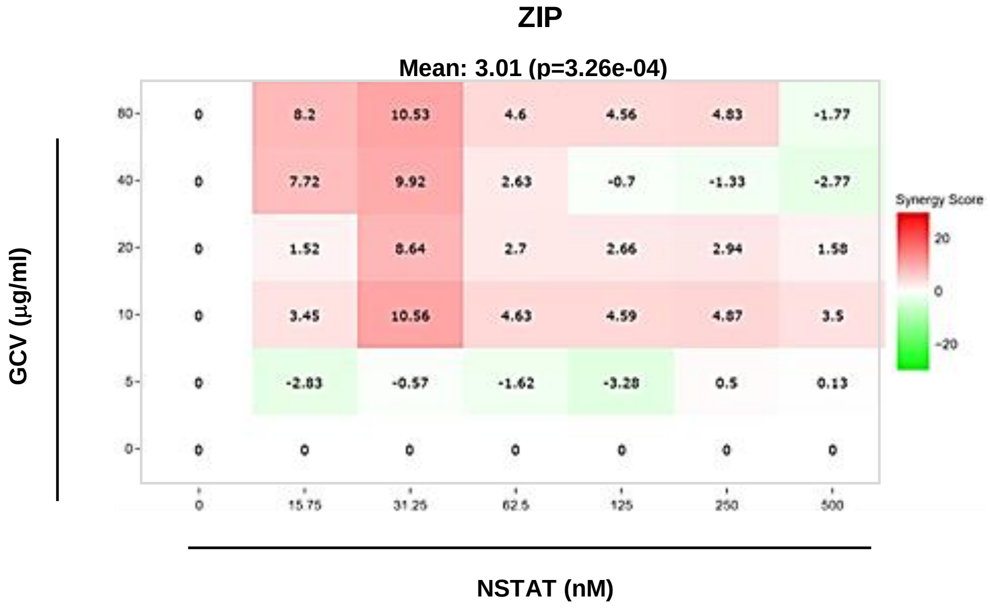

Supplementary Figure S8: SynergyFind 3.0 analysis was used to determine synergistic effects of GCV against SNU719 or YCCEL1 cells treated with various doses of NSTAT.
