# Supplementary material for: Targeting EBV-associated gastric cancer by lytic induction therapy with nanatinostat
Source: Tumour Virus Res. 2026 Jun 27;22:200346. doi: 10.1016/j.tvr.2026.200346 (PMC13332451; doi:10.1016/j.tvr.2026.200346)
Supplement: Multimedia component 2 [file mmc2.pdf]

Genes	Primers for qRT-PCR	Reference
BRLF1	For 5'-GCATGGGCGGGACAATCGCAATATAA -3'<br>Rev 5'-CCAGCCAGATGTTCAGGAACCAAA -3'	[1]
BZLF1	For 5'- TACAAGAATCGGGTGGCTTC -3'<br>Rev 5'- GCACATCTGCTTCAACAGGA -3'	[2]
BMRF1	For 5' - CACTGCGGTGGAGGTAGAG -3'<br>Rev 5'- GGTGGTGTGCCATACAAGG -3'	[1]
BLRF2	For 5' - ACTGAAGCCCAGGACCAGTTCTA -3'<br>Rev 5' - TAAGACAAGCGTCAGAAGTGCCCA -3'	[1]
BGLF4	For 5'- GCTGACTCCACCCACAAAAT -3'<br>Rev 5'- GAGGTCAGGCCCATGTCTAA -3'	[3]
BXLF1	For 5' – AGCTGAAGCGGCTAAGTGGGA -3'<br>Rev 5' - AGGCAGGTTTGATTACTGGTACA -3'	[13]
GAPDH	For 5'- GAAGGTGAAGGTCGGAGTCA -3'<br>Rev 5' – TGACAAGCTTCCGGTTCTC -3'	[4]

References:

[1] Wille, C.K., et al., *5-hydroxymethylation of the EBV genome regulates the latent to lytic switch.* Proc Natl Acad Sci U S A, 2015. **112**(52): p. E7257-65.

[2] Daigle, D., et al., *Valproic acid antagonizes the capacity of other histone deacetylase inhibitors to activate the Epstein-barr virus lytic cycle.* J Virol, 2011. **85**(11): p. 5628-43.

[3] Wildeman, M.A., et al., *Cytolytic virus activation therapy for Epstein-Barr virus-driven tumors.* Clin Cancer Res, 2012. **18**(18): p. 5061-70.

[4] Dabek, J., et al., *Altered transcriptional activity of gene encoding GAPDH in peripheral blood mononuclear cells from patients with cardiac syndrome X - an important part in pathology of microvascular angina?* Arch Med Sci, 2010. **6**(5): p. 709-12.
